# Digital Pathology in South Africa: A Survey and Cross-Sectional Analysis of the Current Level of Use and Comparison With the International Community

**DOI:** 10.1155/ijta/7990652

**Published:** 2025-08-28

**Authors:** Elsamaria Eloff, Renata Schoeman

**Affiliations:** ^1^Department of Leadership, Faculty of Economic and Management Sciences, Stellenbosch Business School, Bellville, South Africa; ^2^Anatomical Pathology, Ampath Laboratories, Pretoria, South Africa

## Abstract

**Background:** Telepathology and digital pathology are increasingly utilised for routine diagnostics globally, improving patient safety and diagnostic efficiency. No research exists on the adoption of digital pathology in South Africa. Understanding the current use can help understand implementation needs and ensure equitable access to this modern diagnostic tool. This can help realise the technology's benefits across the diverse communities in South Africa.

**Methods:** This was a nonexperimental descriptive study. Primary quantitative data were collected via a questionnaire distributed to South African histopathologists in August 2023. The questionnaire focused on participants' demographic information and the use of digital pathology by histopathologists in South Africa. Descriptive data analysis was performed.

**Results:** The study comprised 71 histopathologists, representing 23% of all registered histopathologists in the country. There was a near-equal distribution of female and male histopathologists. Most of the participating histopathologists worked in the private sector. Nearly all of the histopathologists surveyed indicated some exposure to digital pathology (99%). Most participants use digital pathology for external quality assurance and training. The use of digital pathology as a routine diagnostic tool was noted by 22% of participants, although only 3% reported daily use.

**Conclusion:** The current use and adoption of digital pathology by South African histopathologists are comparable to the international community. By increasing the use of digital pathology for routine diagnostic use, we can enhance the accuracy of diagnoses and improve healthcare outcomes for patients and healthcare professionals.

## 1. Introduction

Telemedicine, the electronic transfer of healthcare information and expertise between two geographically separate parties, is a key digital healthcare technology [1], saving time and patient cost in a globally constrained economy [2, 3] and improving healthcare delivery [3–5]. The use of telemedicine has seen considerable growth in the private healthcare sector of South Africa, especially during the COVID-19 pandemic [6, 7], with healthcare workers expressing a general positive attitude to telemedicine, especially in a rural context [8].

Histopathology is well-positioned to benefit from telemedicine, with the rise in the number of cases [9] and the increased demands on report data and quick turnaround time [10, 11]. Telemedicine may be applied to histopathology either in the form of telepathology or digital pathology. Telepathology uses image transmission along with telecommunication links (telediagnosis and teleconsultations) [12], and digital pathology involves a complete digital implementation of the diagnostic process. This includes the digital representation of glass slides, barcode identification and tracking of specimens, image management systems, dictation software, image analysis and computational pathology using artificial intelligence [13–15]. Many studies have explored the potential advantages of digital pathology [16–19]. It has been found to improve safety, efficiency and quality of pathologic diagnoses [20], as well as facilitate collaboration, education and cost savings while supporting the integration of pathology into multidisciplinary teams [11, 12], and expand the geographic reach of pathology [21, 22].

The advantage of expanding geographic reach is particularly important in Africa and South Africa. A recent study counted 108,000 pathologists worldwide [23] at a global average of 14 pathologists per million population. South Africa was found to have 750 practising pathologists, at approximately 12 per million population, while the African average was fewer than three pathologists per million population [23]. In 2021–2022, only 306 histopathologists were registered in South Africa [24]. Digital pathology can, therefore, have a substantial impact on the human resource constraint environment of South Africa and other African countries. Despite this pressing need, studies examining digital pathology and the adoption of digital pathology in South Africa are limited, and to date, no systematic study into the adoption of digital pathology in South Africa has been conducted.

Understanding the current level of adoption and use of digital pathology in South Africa is required to facilitate the wider implementation of this digital health technology with all of its associated benefits. The objective of this study, therefore, is to assess the current level of adoption of digital pathology by South African histopathologists in the private and public sectors and compare this to other countries.

## 2. Methods

The research was based on primary quantitative data collected via a survey distributed to South African histopathologists. It was a purposive sampling of the 306 registered histopathologists in South Africa, with the only inclusion criteria being that participants were qualified histopathologists registered with the Health Professions Council of South Africa to work in South Africa. Histopathologists in both public healthcare and private practice were included, but other pathological disciplines, such as chemical pathologists, hematopathologists, microbiologists and virologists, were specifically excluded. Among a range of digital pathology activities and techniques that may be considered, this study focused on the acceptance of digital pathology in the form of telepathology (as part of email consultations and remote diagnoses), examining WSIs, training, quality assurance programmes and clinical diagnostic work. The use of ancillary technologies such as artificial intelligence and image analysis tools was excluded from the study.

An electronic invitation to participate in the survey was distributed by the South African chapter of the International Academy of Pathologists (IAP) to all South African member histopathologists and via the researcher's LinkedIn social media page. The invitation comprised a brief statement and clarified the purpose of the study. A link was provided so that prospective participants could click to access the electronic questionnaire. The IAP sent the invitation to participate in the research on the researcher's behalf to ensure that the personal information, including the participants' email addresses, remained protected and undisclosed to the researcher to be POPI Act compliant [25].

The total population size was assumed to be 306, as per the latest publicly accessible registration data from 2021 to 2022; the 2022–2023 data was not available at the time of writing [24].

SUNSurveys, a web-based e-survey service hosted by Stellenbosch University, collected the information. The histopathologists had 2 weeks to participate in the study and to complete the questionnaire once it had been launched. The survey tool ensured that the researcher did not have access to any personal identifiers of participants. The questionnaire allowed for the collection of quantifiable data. The data was analysed using IBM's Statistical Package for Social Sciences (SPSS) for Windows Version 28.0 (SPSS, IBM Corp. in Armonk, New York). Illustrative and descriptive statistics were used to analyse the data. All the incomplete questionnaires contained insufficient data for statistical analysis and were not considered.

The questionnaire's content focused on the demographic information of participants and the use of digital pathology by histopathologists in South Africa. It was a structured questionnaire with closed-response questions standardised for all participants. The question–answer formats varied, including yes/no answers and rank-order questions [26, 27]. The demographic information collected included age, gender, nationality, type of laboratory, number of years qualified (an indicator of work experience) and laboratory size. Information about the current use and different applications of digital pathology by the participants, as well as the frequency of use, was also collected.

The research proposal was submitted for ethical clearance to the Stellenbosch Business School Research Ethics Committee, and the study commenced after approval (Project Number 28793).

## 3. Results

A total of 71 completed questionnaires answered by South African histopathologists were received for statistical analysis (*n* = 71). This represents 23% of the 306 registered histopathologists in South Africa and a completion rate of 67.96% of the 103 started questionnaires.

### 3.1. Demographics

A nearly equal distribution of female (35, 49%) and male (36, 51%) histopathologists participated in the study. The mean age of participants was 46 years (SD 10.49 years). Female histopathologists participating in the study were generally younger and were more likely to be between 30 and 40 years (15, 43%) than male histopathologists (10, 28%); however, there was less of a difference in the histopathologists older than 51 years. Most participants were South African (68 of 71, 96%), with a small minority representative of other nationalities. The mean number of years since qualification as a histopathologist was 12 years (SD 7.53 years).  Table 1 provides a summary of the sociodemographic characteristics of the participants. As demographic data of the population of histopathologists is not publicly accessible, it is not possible to assess for potential demographic bias.

### 3.2. Work Environment of Participants

Most participants work at privately owned laboratories (70%), while 30% work at a public National Health Laboratory Service (NHLS) laboratory. On average, approximately 26,000 cases are reported per year per laboratory (mean: 25 985; SD: 14 653) by approximately seven histopathologists (mean: 6.94; SD: 4.05). Female histopathologists were more likely to work in laboratories that reported 20,000 or fewer cases per year (19 of 71, 54%) than male colleagues (12 of 71, 33%).  Table 2 provides a summary of the work environment for participants.

### 3.3. Current Level of Adoption of Digital Pathology by South African Histopathologists

Most South African histopathologists (70, 99%) use an element of digital pathology at least once a year. Participants used digital pathology as part of external quality assurance (EQA) programmes, email consultations, registrar and personal education and routine diagnostic work. An element of digital pathology was used by 41% of participants at least monthly, though the majority of participants (58%) used digital pathology only once a year.

EQA programs were the most frequently used digital pathology modality. Digital pathology use as part of routine diagnostic work was reported by 22% of the participants; however, only 3% of the participants use digital pathology for routine diagnostic work daily. The frequency of use of the different modalities of digital pathology is summarised in Table 3.

When considering the distribution of digital pathology between private and public sector pathologists, only 12% of private pathologists reported daily or weekly use of digital pathology compared to 45% of pathologists in the public sector. Most private pathologists (70%) used digital pathology only once a year, in contrast to publicly employed pathologists who reported more frequent use of digital pathology.

Of the participating histopathologists, 9 (13%) viewed themselves as very knowledgeable about digital pathology. Some knowledge of digital pathology was reported by most participants (55, 77%), whereas only seven (10%) histopathologists said they did not know anything about digital pathology. The seven histopathologists who reported no knowledge of digital pathology were all privately employed histopathologists. All the histopathologists in the public sector reported at least some knowledge of digital pathology, and 20% of public histopathologists reported themselves as very knowledgeable. Only 10% of private histopathologists viewed themselves as very knowledgeable regarding digital pathology.

The expectations of organisations concerning the use of digital pathology by histopathologists were also assessed in the survey, as estimated by the survey participants. Most participants work for organisations that expect them to use digital pathology (54%), but the comparatively lower number of pathologists who do use it for routine diagnostic work (22%) would indicate some barriers to adoption beyond the pathologists' intent evaluated in this study. More than one-third of participants (34%) indicated that they want to use digital pathology regardless of the organisation's expectations, and just under one-third of participants (28%) believe that career progression at their organisation will be facilitated if they use digital pathology.

## 4. Discussion

Our study demonstrated high levels of exposure to digital pathology, where 99% of participants reported using some modality of digital pathology at least once per year. The high percentage of participants reporting experience with digital pathology in South Africa compares well with other international studies and is, in fact, higher than previously reported studies. Numerous international studies have investigated histopathologists' experience with and use of digital pathology. In Switzerland, 89% of pathologists and residents had experience with digital slides, 85% in Canada, 82% in the United States, 66% in India and 60% in the United Kingdom. A recent Saudi Arabian study showed that only 34% of respondents use telepathology [28–32].

Digital pathology was most frequently used in our study as part of an EQA program and secondly for personal educational purposes. This is in comparison to the recent Saudi Arabian study, where only 9% of participants used digital pathology for quality control, and the majority of the participants (86%) used digital pathology for educational purposes. Whether this was for personal education or as part of trainee education was not specified by the Saudi Arabian study [28]. This variation in digital pathology modality between our South African study and the Saudi Arabian study can at least partially be explained by the private–public sector split of participants in our study. Private sector participants in South Africa would generally have no academic commitments, yet would still be expected to participate in quality control programs. The increased frequency of digital pathology use in the public sector in our study could be due to the academic and teaching requirements expected in the public sector, which services tertiary training centres in South Africa. Digital pathology is routinely used in academic teaching [33], and it is the application as a teaching tool that most likely describes its more frequent application in the public sector rather than regular use in clinical practice. Though the percentage of public sector pathologists routinely using digital pathology is more than in the private sector, these results should be interpreted with caution as only 30% of our study participants were employed in the public sector. This is in comparison to the Saudi Arabian study, which comprised pathology departments in the Ministry of National Guard Health Affairs (MNGHA) hospitals in Saudi Arabia, a more uniform work environment concerning academic commitments [28].

The reported overall use of digital pathology for routine diagnostic work in South Africa (22%), as illustrated in the current study, is comparable to that of the international community. The United Kingdom reported that 31% of histopathologists use digital pathology for primary diagnostic work, and 36% of respondents use it for secondary diagnosis [32]. In Switzerland, more than a fifth of the respondents (22%) indicated using digital pathology for primary diagnostic work. The Swiss study also noted that just over half of the participants use digital pathology at least monthly (20%) or weekly (31%). The Swiss study, therefore, showed that digital pathology was more frequently used by more of the participants in comparison to our study, where only 41% of the participants used digital pathology on at least a monthly basis [30]. Weekly use of digital pathology for routine diagnostic work was reported by 11% of participants in our study, and only 3% of the participants reported daily use of digital pathology for routine diagnostic work. This discrepancy between the number of histopathologists who use digital pathology for diagnostic purposes in general and those who do so daily could be due to a hybrid work setup of laboratories in South Africa during the transition from light microscopy to a digital workflow. This indicates that digital pathology has seen some adoption in South Africa, but that even among pathologists who have adopted it partially, it is still not the sole or main modality of viewing slides and that the frequency of routine use lags behind the international community.

The study from India noted a higher percentage of participants who use digital pathology for routine diagnostics as compared to the 22% of participants reported in our study. In the Indian study, 28% of histopathologists use digital pathology for diagnostic work (routine and challenging cases). Fewer participants in the Saudi Arabian study (14%) reported using digital pathology for primary diagnostic work [28, 29]. The studies did, however, not specify the frequency of use for routine diagnostic work as was stipulated in our research.

The current level of exposure to and adoption of digital pathology in South Africa is comparable to that of developed countries. The higher level of overall exposure to digital pathology in the current study could be because most other studies were performed more than 5 years ago in a rapidly developing field.

There are several limitations of this study that should be considered, chief among these is the small sample size. Though nearly a quarter of the registered histopathologists in South Africa responded to the survey, a larger sample size would allow for more nuanced information regarding the current level of digital pathology adoption in South Africa. Potential bias considerations included selection bias, nonresponse bias and social desirability bias. A factor that might influence selection bias is that inclusion in the study required IAP membership or a presence on LinkedIn, although neither is required to be a practising histopathologist in South Africa. As there are no detailed demographics of the population publicly available, participation bias could not be evaluated [34]. The IAP membership directory is, however, the most extensive collection of histopathologists in South Africa, and its use mitigated the potential bias of recruitment on social media alone. A better-represented sample of publicly employed histopathologists (versus participants from private practice) could offer new insights regarding the level of adoption and use of digital pathology in the public sector.

There was a risk of nonresponse bias as histopathologists who work in high-throughput laboratories do not have the time to complete a lengthy questionnaire. This was addressed by limiting the length of the questionnaire and making it as quick and easy as possible to complete. Social desirability bias was mitigated because of the anonymous nature of an online questionnaire.

Future research with a larger sample size and improved representation of publicly NHLS-employed histopathologists is suggested. Additional future research could also investigate the process of organisations transitioning from light microscopy to a digital workflow and the barriers and enablers of this process. This could provide insight into some of the interesting discrepancies noted in this study with regard to the low numbers of histopathologists who daily use digital pathology for diagnostic work, as well as organisational expectations that exceed actual usage of digital pathology.

## 5. Conclusion

This study provides the first assessment of digital pathology adoption in South Africa. While nearly all histopathologists reported some exposure to digital pathology, routine diagnostic use remains limited, with only 22% incorporating it and just 3% using it daily. The predominant application is in EQA programmes rather than routine diagnostics, limiting its full potential.

## Figures and Tables

**Table 1:** Sociodemographic characteristics of participants (*n* = 71).

**Measure**	**Item**	**Count (** **n** = 71**)**	**Percentage**
Gender	Female	35	49%
	Male	36	51%

Age	30–40 years	24	34%
	41–50 years	26	37%
	51–60 years	10	14%
	> 60 years	11	15%

Nationality	South African	68	96%
	Other	3	4%

Number of years qualified	< 5 years	20	28%
	5–10 years	12	17%
	11–15 years	13	18%
	16–20 years	7	10%
	> 20 years	19	27%

**Table 2:** The work environment of the participants (*n* = 71).

**Measure**	**Item**	**Count (** **n** = 71**)**	**Percentage**
Type of laboratory	Private	50	70%
	Public/NHLS	21	30%

Number of cases/year	< 10,000	11	15%
	10,001–20,000	21	30%
	20,001–30,000	7	10%
	30,001–40,000	14	20%
	> 40,000	18	25%

Number of histopathologists	< 5	30	42%
	5–10	28	39%
	10–15	11	15%
	> 15	2	3%

**Table 3:** Frequency of use of the different digital pathology modalities by the study participants (*n* = 71).

**Activity**	**Never**	**At least once a year**	**Monthly**	**Weekly**	**Daily**
External quality assurance program	1 (1%)	50 (70%)	18 (25%)	2 (3%)	0 (0%)
Email consultation with attached photographs	37 (52%)	25 (35%)	5 (7%)	4 (6%)	0 (0%)
Registrar training	39 (55%)	13 (18%)	11 (15%)	8 (11%)	0 (0%)
Routine diagnostic work	55 (77%)	6 (8%)	0 (0%)	8 (11%)	2 (3%)
Personal education	14 (20%)	25 (35%)	16 (23%)	11 (15%)	5 (7%)

## Data Availability

The dataset underlying the results presented in this study may be made available upon request from the corresponding author, Dr Elsamaria Eloff, at eloffe@ampath.co.za.
